# Optimized Derivation of Predicted No-Effect Concentrations (PNECs) for Eight Polycyclic Aromatic Hydrocarbons (PAHs) Using HC_10_ Based on Acute Toxicity Data

**DOI:** 10.3390/toxics11070563

**Published:** 2023-06-28

**Authors:** Xiao Sun, Ting-Ting Ding, Ze-Jun Wang, Peng Huang, Shu-Shen Liu

**Affiliations:** 1 Key Laboratory of Yangtze River Water Environment, Ministry of Education, College of Environmental Science and Engineering, Tongji University, Shanghai 200092, China; sunx9325@163.com (X.S.); ding_tingting@126.com (T.-T.D.); huangpeng425@163.com (P.H.); 2 State Key Laboratory of Pollution Control and Resource Reuse, College of Environmental Science and Engineering, Tongji University, Shanghai 200092, China; 3 Shanghai Institute of Pollution Control and Ecological Security, Shanghai 200092, China; 4 National and Local Joint Engineering Laboratory of Municipal Sewage Resource Utilization Technology, School of Environmental Science and Engineering, Suzhou University of Science and Technology, Suzhou 215009, China; wangzj9135@163.com

**Keywords:** two-parameter nonlinear functions, cumulative probability, toxicity on multi-species, median effect concentration, no observed effect concentration, aquatic organisms

## Abstract

For persistent organic pollutants, a concern of environmental supervision, predicted no-effect concentrations (PNECs) are often used in ecological risk assessment, which is commonly derived from the hazardous concentration of 5% (HC_5_) of the species sensitivity distribution (SSD). To address the problem of a lack of toxicity data, the objectives of this study are to propose and apply two improvement ideas for SSD application, taking polycyclic aromatic hydrocarbons (PAHs) as an example: whether the chronic PNEC can be derived from the acute SSD curve; whether the PNEC may be calculated by HC_10_ to avoid solely statistical extrapolation. In this study, the acute SSD curves for eight PAHs and the chronic SSD curves for three PAHs were constructed. The quantity relationship of HC_5_s between the acute and chronic SSD curves was explored, and the value of the assessment factor when using HC_10_ to calculate PNEC was derived. The results showed that, for PAHs, the chronic PNEC can be estimated by multiplying the acute PNEC by 0.1, and the value of the assessment factor corresponding to HC_10_ is 10. For acenaphthene, anthracene, benzo[a]pyrene, fluoranthene, fluorene, naphthalene, phenanthrene, and pyrene, the chronic PNECs based on the acute HC_10_s were 0.8120, 0.008925, 0.005202, 0.07602, 2.328, 12.75, 0.5731, and 0.05360 μg/L, respectively.

## 1. Introduction

Polycyclic aromatic hydrocarbons (PAHs) are a class of neutral or non-polar hydrocarbons formed by the linear, angular, or cluster-like linkages of two or more benzene rings [1]. More than 200 PAHs are widely distributed in various environments [2], the main sources of which are the incomplete combustion of organic matter such as fossil and biomass fuels [3] and rock-forming processes [4]. Due to surface runoff, atmospheric deposition, and wastewater discharge [5], PAHs are widely distributed in water bodies all over the world, with concentrations ranging from μg/L to ng/L. There have been many studies analyzing the distribution, source, and risk associated with PAHs in aquatic environments from different areas [6,7,8,9,10]. PAHs exhibit teratogenic and carcinogenic properties, bioaccumulation, and long-range transport properties, considered to be persistent in the environment [2,11]. There are studies that have reviewed and summarized the hazardous effects caused by PAHs on different aquatic organisms [12,13], including fish [13], algae [14], and benthic fauna [1]. PAHs have drawn regulatory attention. The U.S. Environmental Protection Agency (US EPA) has listed 16 PAHs as priority control pollutants [1], while the Scientific Committee on Food (SCF) has identified 15 PAHs possessing both genotoxic and carcinogenic properties [15].

When completing an ecological risk assessment and creating water quality standards, the predicted no-effect concentration (PNEC) is frequently utilized. If the predicted environmental concentration is lower than the PNEC, the ecological risk of the chemical is generally considered acceptable [16]. There are two major approaches to deriving PNECs: a deterministic approach based on the use of an assessment factor (AF) and a statistical approach based on species sensitivity distribution (SSD) [16,17]. The AF method can be applied to any sample size. In the AF technique, the PNEC is derived by dividing the lowest value of qualified toxicity data (e.g., LC_50_, EC_50_, NOEC) by an appropriate AF [18]. There are a variety of values for AF, including 10, 50, 100, and 1000, depending on the amount and quality of the toxicity data, such as long-term or short-term data, and how many trophic levels are included [19]. If a large data set from different taxonomic groups is available, the SSD method is often used. As a statistical extrapolation method, SSD is based on a cumulative probability (CP) distribution. The main assumptions underlying SSD are as follows: (1) the distribution of species sensitivities follows a theoretical distribution function; and (2) the group of species tested in the laboratory is a random sample of this distribution [19]. Essentially, the SSD method assembles single-species toxicity data to predict a hazardous concentration (HC) that affects a certain percentage (p) (HC_p_) of all the species in a distribution [20], and the HC_p_ also needs to be divided by an AF ranging from 1 to 5 to achieve the protection goal [16]. Compared with the AF method, the advantages of the SSD method are as follows: (1) the reliability of risk assessment is possible to quantify and know owning to the confidence intervals; (2) the data utility is effectively improved [16]; and (3) the whole sensitivity distribution of species in an ecosystem is used instead of the lowest toxicity data [19]. Following its introduction in the 1980s, the SSD has remained the most widely used method for deriving water quality benchmarks to characterize the effects of chemical contaminants on water quality [21]. In recent years, various published research and reviews have aimed at improving SSD methods [20,22,23]. Compared with acute toxicity data, chronic toxicity data are more ecologically realistic and can more accurately reflect the long-term impacts of substances on organisms [24]. No observed effect concentration (NOEC) is a commonly used toxic measurement in SSD estimation, and it has also been discovered that EC_10_ can be taken into account as an equivalent to NOEC in SSD construction [25]. However, chronic toxicity tests are more expensive and time-consuming, and the majority of substances, including PAHs, have little long-term toxicity data [26]. Based on acute toxicity data, several studies have built the SSD curves for PAHs [1,27,28,29,30]. When there is insufficient chronic toxicity data, it is crucial to know how to obtain chronic PNEC. One situation is that the chronic toxicity data are not enough to create the SSD curve, but can be supplemented by some methods. For PAHs, there is research using chronic toxicity data in both freshwater and saltwater to develop SSD curves [31], but it is important to think more carefully before combining data from various exposure media. Transforming acute data into chronic data can supply chronic data; at the moment, the acute-to-chronic ratio (ACR) method is a common conversion method [32]. The ACR method should be used in conjunction with the acute and chronic toxicity data for at least three species, including one fish, one invertebrate, and another aquatic organism [33]. Another circumstance is when the chronic toxicity data is totally null or does not conform to the requirements of the data supplement methods (e.g., ACR). Default values of various orders of magnitude (e.g., 10, 100, 1000) are used in some regulatory documents, such as the guidelines within Registration, Evaluation, Authorization, and Restriction of Chemicals (REACH) [34] and the Organization for Economic Co-operation and Development (OECD) [35]. In addition, the ACRs of algae (33.3), invertebrates (41.4), and vertebrates (198.2) suitable for more than 90% of toxic substances were also used to provide chronic data and compute PNEC for PAHs [24]. However, the reactions of different species to the chemicals are usually different, leading to different ACRs. Directly using the default or literature values of ACR for PAHs to obtain the chronic toxicity data for different chemicals in different species may cause the SSD curves to integrally shift, leading to an unsuitable ecological threshold that causes over-protection or under-protection. Therefore, how to derive long-term PNEC from short-term toxicity data is a problem to be solved, concerning the relationship between the HC_5_s of the acute and chronic curves. In the research of Hiki and Iwasaki [36], on the basis of a comprehensive analysis of 150 pairs of acute and chronic SSD curves for 150 chemicals, it was proposed that multiplying by a factor of 0.1 to obtain a first approximation of the chronic HC_5_ from the acute HC_5_ is defensible, and multiplication by a factor of 0.01 can provide a conservation HC_5_ covering 134 out of 150 chemicals. This provides insight into how chronic PNEC can be derived from acute data.

For PNEC determination using the SSD method, the hazardous concentration (HC_5_) based on the SSD curve’s 5% CP is typically used [37]. When toxicity data are insufficient, the sensitivity of HC_5_ to the species in the left tail of the SSD curve is a significant issue. According to the commonly used formula: CP = i/*n* + 1 [24,32], where i is the ascending order number of toxicity data and *n* is the number of toxicity data, there is no data point under 5% CP if the amount of data is less than 19, indicating that the HC_5_ is calculated from statistical extrapolation with high uncertainty. An example is the SSD curves for nickel constructed by DeForest and Schlekat [38] using the toxicity data of 17 marine organisms, in which the HC_5_ values obtained were 3.9 and 20.9 mg/L with and without the most sensitive species *Diadema antillarum*. However, the lack of toxicity data is a prevalent issue in the construction of SSD curves for various chemicals [39], including PAHs [24,27,28,40]. HC_5_ with a high degree of uncertainty will result in an uncertain PNEC; therefore, it deserves thought to increase CP when calculating hazardous concentrations using the same data to reduce uncertainty. For instance, if the CP is increased from 5% to 10%, the minimal number of species required for at least one data point under 10% CP is reduced from 19 to 9 [41]. The expected 95% protection level remains unchanged, and the PNECs from HC_5_ and HC_10_ are supposed to be uniform. How to determine the value of the assessment factor for HC_10_ when that for HC_5_ usually ranges from 1 to 5 presents a problem [16]. The correlation between HC_5_ and HC_10_ needs to be determined in order to determine the assessment factor for HC_10_. There is research deriving the assessment factor for HC_10_ based on the 35 SSD curves for pesticides [41], but whether it is suitable for other chemicals such as PAHs needs further research.

The objectives of the present work are to explore, for PAHs, how to derive the chronic PNEC from the acute SSD curves and calculate the PNEC by HC_10_, and finally, put it into practice by deriving the chronic PNECs from the acute HC_10_s to improve the derivation results. Eight PAHs that were prevalent in water were studied in this research, namely acenaphthene (ACE), anthracene (ANT), benzo[a]pyrene (B[a]P), fluoranthene (FLA), fluorene (FLO), naphthalene (NAP), phenanthrene (PHE), and pyrene (PYR). The acute (LC_50_ or EC_50_) and chronic (NOEC or EC_10_) toxicity data for PAHs in a variety of species were obtained by searching multiple databases and the published literature. The main contents are as follows: for PAHs, (1) the optimal SSD models were developed based on acute and chronic toxicity data using multiple two-parameter nonlinear sigmoid functions; (2) the quantity relationship between the acute and chronic SSD curves was discussed to determine the calculation factor from the acute to chronic PENCs; (3) the value of AF corresponding to HC_10_ when deriving PNEC was determined; and (4) the chronic PNECs from the HC_10_ on the acute SSD curves for eight PAHs were derived with improvement in reliability.

## 2. Materials and Methods

### 2.1. Acquisition, Screening, and Processing of Toxicity Data

Eight PAHs, namely ACE (CAS: 83-32-9), ANT (CAS: 120-12-7), B[a]P (CAS: 50-32-8), FLA (CAS: 206-44-0), FLO (CAS: 86-73-7), NAP (CAS: 91-20-3), PHE (CAS: 85-01-8), and PYR (CAS: 129-00-0), were chosen as research objects to derive PNECs. The method of data screening and preprocessing was determined following the principles of appropriateness, accuracy, and reliability, as well as a technical guidance document on risk assessment [19]. Through a multiple-source data search, including databases such as USEPA ECOTOX (https://cfpub.epa.gov/ecotox/search.cfm), (accessed on 7 January 2023) USEPA pesticide data, (http://www.epa.gov/pesticides), (accessed on 7 January 2023) the Environ database (https://envirotoxdatabase.org/), (accessed on 7 January 2023) eChemPortal (https://www.echemportal.org/echemportal/), (accessed on 7 January 2023) and literature libraries (https://www.cnki.net/ and http://www.sciencedirect.com), (accessed on 7 January 2023) the toxicity data of these eight PAHs in freshwater media were gathered. The species groups covered were algae, amphibians, crustaceans, fish, insects, invertebrates, mollusca, plants, and worms.

As for acute toxicity data for PAHs, the median effect concentration (EC_50_) and median lethal concentration (LC_50_) were used as measurement endpoints. For chronic toxicity data, no observed effect concentration (NOEC) was the first choice, but 10% effective concentration (EC_10_) was used when NOEC was unavailable [25]. Typically, data lacking detailed information regarding exposure type, exposure durations, endpoints, and effects were excluded. There are static, renewal, and flow-through exposure types. The durations were referred to OECD guidelines for the test chemicals (https://doi.org/10.1787/20745761), (accessed on 7 January 2023) US EPA ecological effects tests (https://www.epa.gov/test-guidelines-pesticides-and-toxic-substances/series-850-ecological-effects-test-guidelines), (accessed on 7 January 2023) and ATM Environmental toxicology standards (https://www.astm.org/products-services/standards-and-publications/standards/environmental-toxicology-standards.html), (accessed on 7 January 2023). The definitions of acute and chronic toxicity vary by program and the tested organism, with a greater emphasis on exposure duration for acute and chronic data that can be significantly differentiated. Acute tests generally lasted no more than 96 h; for vertebrates, including fish and amphibians, it was 96 h and for invertebrates and algae it was 24 to 96 h. In general, chronic tests lasted no less than 10 days, and, in most cases, no less than 21 days. If there were multiple values of one acute or chronic toxicity endpoint for one species for a given PAH, the geometric mean was calculated, and the most sensitive acute or chronic endpoint was retained for each species. The sample size (number of test species) required to estimate SSDs is dependent on the regulatory jurisdiction, and various values have been proposed, which typically range from 5 to 10 [20]. In this study, the minimum requirement for a sample size was eight.

The ACR method was utilized to convert the acute toxicity data of B[a]P, FLA, FLO, and PHE into chronic ones. The acute and chronic toxicity data for at least one fish, one invertebrate, and one additional aquatic organism for each PAH were utilized to calculate species acute-chronic ratios (SACRs). The geometric mean of each SACR of one PAH was calculated as its final acute-to-chronic ratio (FACR).

### 2.2. The Construction of SSD Curves

To begin with, toxicity data (acute or chronic) were ascendingly sorted, and cumulative probability (CP) was calculated according to Equation (1).

(1) $$\mathrm{CP}=\frac{i}{n+1}$$

where *i* is the ascending order number of toxicity data (*x*) and *n* is the total number of toxicity data. To identify the optimal SSD fitting functions for PAHs, nine two-parameter nonlinear functions [41] were chosen to fit the (*x*, CP) data. The equations of nine functions are shown in Table S1. The optimal fitting models were determined using the determination coefficient (*R*^2^) and the root mean squared error (RMSE) of the data below 50% CP, and the 95% observation-based confidence intervals (OCIs) were calculated [42]. In contrast to computing the RMSE of the entire curve, the RMSE of the data below 50% CP, i.e., RMSE_50_, provides a more accurate reflection of the degree of fitting at the lower end of the curve (closer to HC_5_ or HC_10_), thereby ensuring the confidence level of the calculated hazardous concentration of low CP, such as HC_5_ and HC_10_ [43].

### 2.3. The Calculation of PNECs

Based on the optimal SSD models found in Section 2.2, two hazard concentrations (HC_5_ and HC_10_) were calculated. The PNECs for PAHs were calculated according to Equations (2) or (3):

(2) $$\mathrm{PNEC}=\frac{{\mathrm{HC}}_{5}}{{\mathrm{AF}}_{5}}$$

(3) $$\mathrm{PNEC}=\frac{{\mathrm{HC}}_{10}}{{\mathrm{AF}}_{10}}$$

where AF_5_ is the assessment factor based on HC_5_, ranging from 1 to 5 [16], and AF_10_ is the assessment factor based on HC_10._ In this study, AF_5_ was conservatively taken as 5. Given that, at a 95% protection level, the PNECs derived from different methods for a PAH should not significantly differ, the AF_10_, different from the AF_5_, should be used when calculating a PNEC based on HC_10_. The fundamental mathematical equation relation is that “PNEC = HC_5_/AF_5_ = HC_10_/AF_10_”. Considering that the values of HC_5_ and HC_10_ must be reliable before calculating AF_10_, SSD curves with data points below 5% CP are selected to compute each individual AF_10_, and the geometric mean of each individual AF_10_ is the final AF_10_.

The construction of SSD models and confidence intervals, the calculation of statistics *R*^2^ and RMSE_50_, the acquisition of HC_5_ and HC_10_ by inverse functions, the estimation of PNECs, and other data processing work associated with this study were performed on the software platform mPNEC (Environmental Pollution Mixture PNEC Calculation Software, computer software copyright registration certificate No. 04615136, registration number 2019SR1047553) [43], which was independently developed by our research group.

## 3. Results and Discussion

### 3.1. The Optimal Fitting of SSD Curves

The collected acute toxicity data for eight PAHs and the chronic toxicity data for four PAHs are shown in Tables S2–S9, including the endpoints, exposure duration, and exposure type. The aquatic organisms covered by the toxicity data are shown in Table S10. ACE, ANT, B[a]P, FLA, FLO, NAP, PHE, and PYR have LC_50_ or EC_50_ values for thirteen, ten, twenty-one, thirty-one, ten, twenty-five, twenty-nine, and eleven species, respectively (Table 1), and B[a]P, FLA, FLO, and PHE have NOEC or EC_10_ values for eight, ten, five, and nine species, respectively (Table 1). SSD curves were constructed using the acute data for eight PAHs and the chronic data for three PAHs, with the exception of the chronic data for FLO, with only five species.

**Figure 1:**
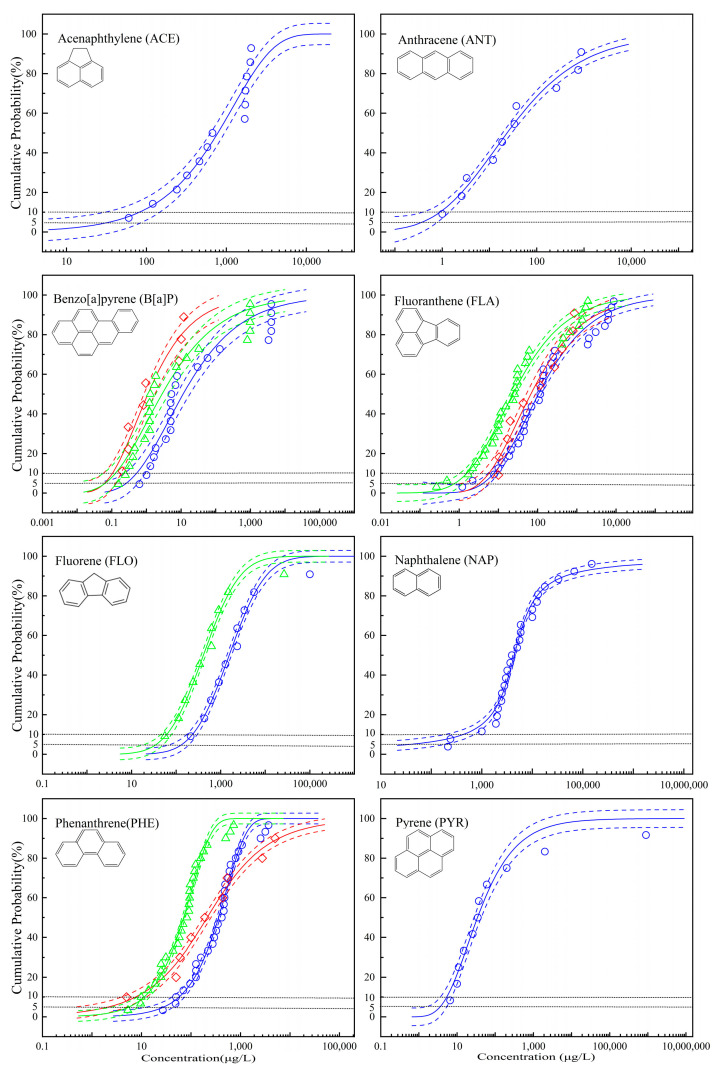
The AV-based (◯), CV-based (◇), and CV_ES_-based (△) SSD curves (blue, red, and green) of various polycyclic aromatic hydrocarbons (PAHs), respectively, where AV refers to the acute toxicity value, CV to the chronic toxicity value observed, and CV_ES_ to the chronic value estimated by the acute-to-chronic ratio (ACR) method.

According to the method in Section 2.2, the acute toxicity data for eight PAHs and the chronic toxicity data for three PAHs were sorted, and the CP data were calculated. The toxicity-CP data were fitted with nine two-parameter nonlinear fitting functions (Table S1), with the results presented in Tables S11 and S12. Using *R*^2^ and RMSE_50_ as optimization objectives, the optimal SSD models of PAHs were determined. Table 2 displays the regression coefficients (*a* and *b*) and fitting statistics (*R*^2^ and RMSE_50_) of eight SSD models based on acute toxicity data and three SSD models based on chronic toxicity data. The acute SSD curves (blue) for all eight PAHs and the chronic SSD curves (red) for three PAHs (B[a]P, FLA, and PHE) are shown in Figure 1. The green triangles and green curves in Figure 1 represent the estimated chronic SSD curves by the ACR method (details in Section 3.2).

Except for the optimal acute SSD curves of ACE (*R*^2^ = 0.9284) and B[a]P (*R*^2^ = 0.9301), and the optimal chronic SSD curve of B[a]P (*R*^2^ = 0.9240), all other optimal SSD curves had *R*^2^ values greater than 0.95, indicating a good overall fit. The fitting functions commonly used in SSD include the normal, logistic, and burr type III functions [24,26], and in this study, nine fitting functions (Table S1), including normal and logistic functions, were chosen for comprehensive analysis to identify the optimal functions for eight PAHs. According to the results in Table 2 and Figure 1, the optimal SSD models for different PAHs are not uniform, and the optimal SSD models based on the acute and chronic toxicity data for the same PAH are also not the same. All optimal SSD models incorporate weibull, error, gompertz, dagum, and guidermannian functions, with weibull and gompertz functions being the most frequently selected. This demonstrates that there is no absolute optimal function for SSD model building using toxicity data from various species for various chemicals and that it is necessary to simultaneously implement and compare multiple fitting functions. Model averaging is one method for integrating the outcomes of multiple functions [22]. In brief, by using the maximum likelihood methods to fit the candidate models, the weight of each model is calculated based on the information-theoretic value (e.g., the Akaike information criterion) of every candidate fit, and then the estimated value is computed by the model weights [22]. Model averaging can be used to retain information obtained from multiple distributions, but some issues need further research, such as the determination of weight needs to be very cautious and whether functions with poor fitting effects need a non-zero weight to act as part of the final value. In this study, the statistics *R*^2^ and RMSE_50_ were used in conjunction to determine the optimal fitting function for each PAH. It is important to note that RMSE_50_ provides a more accurate reflection of the degree of fitting at the lower end of the curve, thereby helping to ensure the reliability of the calculated hazardous concentration of low CP. Some studies have applied the nine fitting functions used in this study to construct the SSD models, and held similar views [41,43]. Regardless, there is no universal fitting function pertinent to SSD models for different chemicals, and multiple fitting functions should be utilized and treated.

### 3.2. The Feasibility of Using Acute Toxicity Data to Derive Chronic PNECs

In general, it is believed that PNECs derived from chronic toxicity data are more reflective of the practical environment. However, only a few chemicals have sufficient chronic toxicity data, and the majority lack long-term data [26]. In the present study, the chronic SSD models of only three PAHs (B[a]P, FLA, and PHE) with no less than eight chronic toxicity data points were constructed. For the other five PAHs, only FLO had chronic toxicity data from five species, while the other four PAHs had no chronic data. How to use existing acute data to construct SSD models and derive long-term PNECs without conducting chronic toxicity testing is an important issue. The ACR method is effective in converting the acute toxicity value (AV) to the chronic toxicity value (CV) as a supplement to the chronic data for specific chemicals in specific species [26]. In this study, the SACRs of four PAHs were calculated (AV/CV), namely B[a]P, FLA, FLO, and PHE, and the geometric means of the SACRs for each PAH on multiple species were calculated as the FACRs (Table 3); these FACRs were then used to convert the AVs of PAHs to the estimated chronic toxicity values (CV_ES_s).

The CV_ES_s of B[a]P, FLA, FLO, and PHE were arranged in ascending order, and the corresponding CPs were calculated. The SSD models based on the CV_ES_s were created using the same method (Table S12). The optimal SSD curves are shown in Figure 1 (green curves), and the results of fitting are provided in Table 2. For B[a]P and PHE, the green curves (CV_ES_) and the red curves (CV) are close to each other at low CP. The ratios of HC_5_ (CV) to HC_5_ (CV_ES_) for B[a]P, FLA, and PHE range from 0.38 to 4.26. The means and standard deviations (SDs) of log_10_-transformed CVs and log_10_-transformed CV_ES_s for B[a]P, FLA, and PHE were calculated and compared, respectively. The difference between the mean of log_10_-transformed CVs and CV_ES_s ranges from −0.65 to 0.52, and the difference between the SD of log_10_-transformed CVs and CV_ES_s ranges from −0.61 to 0.40. The details regarding means and SDs are shown in Table S13. In addition, a two-sample Kolmogorov-Smirnov (K-S) test was also used to compare SSD curves. The results of the K-S test showed that there was no significant difference between the distributions of chronic and ACR-transformed data (*p >* 0.05) for B[a]P (*n*1 = 8, *n*2 = 21, *p* = 0.4123), FLA (*n*1 = 10, *n*2 = 31, *p* = 0.2176), and PHE (*n*1 = 9, *n*2 = 29, *p* = 0.1313). Taking all comparison results into account, it is thought that the ACR method can be used to supply chronic toxicity data when using the SSD model to calculate PNEC. Similar treatment was applied to FLO with insufficient CVs, and the SACR values for each species and the FACR value are listed in Table 3. The SSD model based on the CV_ES_s of FLO was also developed (Table 2), and the optimal SSD curve is shown in the green curve of FLO in Figure 1.

ACE, ANT, NAP, and PYR lacked chronic toxicity data, which means that the ACR method is inapplicable. It merits consideration whether chronic PNEC can be directly derived from SSD curves based on AVs. Analyzing the quantitative relationship between the acute and chronic SSD curves of a large number of chemicals from a holistic perspective is practical in order to find a summarized rule that can be applied to other chemicals lacking chronic data. In the study by Hiki and Iwasaki [36], 150 pairs of acute and chronic SSD curves for 150 chemicals were constructed. The critical results were as follows: (1) on average, the means of log_10_-transformed chronic toxicity data were approximately ten times lower than those of acute data, and for many chemicals, the ratios of chronic to acute data means ranged from 0.01 to 1; (2) the SDs of log_10_-transformed acute data closely overlapped those of chronic data; (3) multiplying by a factor of 0.1 to obtain a first approximation of the chronic HC_5_ from acute HC_5_ is defensible, and multiplication by a factor of 0.01 can provide a conservation HC_5_ covering the HC_5_s of 134 out of 150 chemicals. There was no significant difference between the ratios of the mans or SDs of log_10_-transformed chronic to acute toxicity data among the three modes of action (narcotic, specifically acting, and unclassified). Although the absolute of the ratios of chronic to acute means decreased as the number of tested species increased, they always fluctuated within a range with 10 as the center, 100 as the upper limit, and 1 as the lower limit. In conclusion, it appears that the chronic HC_5_ can be estimated by multiplying the acute HC_5_ with 0.1 for chemicals lacking chronic data; thus, the acute and chronic SSD curves for PAHs constructed in this study were examined. The means and SDs of log_10_-transformed AVs for B[a]P, FLA, FLO, and PHE, and log_10_-transformed CV_ES_s for FLO were calculated. For B[a]P, FLA, and PHE, (1) the ratios of HC_5_ (AV) to HC_5_ (CV) are 4.24, 1.23, and 13.26, respectively; (2) the difference between the means of log_10_-transformed AVs and CVs is 1.26, 0.13, and 0.19, respectively; and (3) the difference between the SDs of log_10_-transformed AVs and CVs is 0.61, 0.31, and −0.40, respectively. For FLO, the ratio of HC_5_ (AV) to HC_5_ (CV_ES_) is 3.79, and the difference between the means of log_10_-transformed AVs and CV_ES_s is 0.58. The details regarding means and SDs are shown in Table S13. Although the ratio of acute to chronic HC_5_ for PHE is greater than ten, i.e., 13.26, it is close to ten. The results of the K-S test showed that the distribution of chronic and acute data for FLA (*n*1 = 31, *n*2 = 10, *p* = 0.5821) and PHE (*n*1 = 29, *n*2 = 9, *p* = 0.5883) had no significant difference (*p* > 0.05), except for B[a]P (*n*1 = 21, *n*2 = 8, *p* = 0.0238). It is notable that the acute SSD curve for FLA is nearly overlapping with its chronic SSD curve, presenting a very small distance between them. Therefore, the chronic HC_5_ can be approximated by multiplying the acute HC_5_ with a factor of 0.1 for PAHs. On the basis of the relationship between acute and chronic HC_5_s, the relationship between acute and chronic PNECs was hypothesized. According to Equation (2), in which the maximum value 5 of AF_5_ is used as the default value, if the value of AF_5_ is unchanged, the ratio of the chronic PNEC to the acute PNEC is equal to the ratio of the chronic HC_5_ to the acute HC_5_; i.e., 0.1. Therefore, when using HC_5_ to calculate PNEC, the chronic PNEC can be estimated by multiplying the acute PNEC by 0.1.

### 3.3. Derivation of Chronic PNECs for Eight PAHs Using HC_10_ to Reduce Uncertainty

The confidence degree of the calculated HC_5_ is significantly impacted by the sensitive species at the end of the curve (low CP area). Specifically, no data points are fitted in the region with a CP of 5% or less when the toxicity data are insufficient, and the HC_5_ fully comes from statistical extrapolation, which increases the uncertainty of the HC_5_ value and then the PNEC. As depicted in Figure 1, there are no experimental observation data points at or below 5% CP in the acute SSD curves of ACE, ANT, FLO, and PYR, as well as the chronic SSD curves of B[a]P, FLA, and PHE. Existing studies that utilized SSD to determine the environmental criteria for PAHs encountered the same issue [24,28,31,44], but the SSD curves constructed in most studies have at least one data point below 10% CP. In Section 3.2 of the present study, it is suggested that one-tenth of the acute PNEC can be used to estimate the chronic PNEC, and all eight acute SSD curves have data points below 10% CP. Therefore, appropriately increasing CP to 10% when calculating the hazardous concentration is helpful to reduce its uncertainty and the uncertainty of the derived PNEC. It is important to note that the expected protection level of 95% remains the same regardless of the hazardous concentration used to calculate the PNEC.

AF_5_, which corresponds to PNEC derived from HC_5_, usually ranges from 1 to 5 [16], and a conservative estimate of 5 is used in this study. According to the principle that, with an unchanged protection level of 95%, the determined chemical should have the determined PNEC, the derivation of PNEC based on HC_10_ requires suggesting an AF_10_ that is different from AF_5_. The key mathematical relationship is that the ratio of HC_5_ to AF_5_ is equal to the ratio of HC_10_ to AF_10_, where AF_5_ equals 5. The values of HC_5_ and HC_10_ should be reliable before calculating AF_10_; therefore, four SSD curves with more than 19 species data are chosen to calculate each individual AF_10_, namely the acute SSD curves of B[a]P, FLA, NAP, and PHE. Then, the geometric mean of the four AF_10_s is calculated as the final AF_10_. The AF_10_s of B[a]P, FLA, NAP, and PHE are 9.89, 9.30, 6.55, and 10.08, respectively, and the final AF_10_ is 8.83, and is conservatively estimated to be 10. This is inconsistent with the recommended value of 50 of AF_10_ for pesticides [41], which is based on the SSD curves of 35 pesticides. The variation in AF_10_ results is partly attributable to the diverse species used to develop the SSD curves for pesticides and PAHs, and PAHs and pesticides have different physical and chemical properties. The verification of the accuracy and rationality of the derivation method of AF_10_ requires more research on water quality criteria by HC_10_. It is currently recommended to specifically derive the appropriate AF_10_ for different compounds.

Table 4 lists the acute and chronic PNECs for eight PAHs based on HC_10_ and HC_5_. The PNECs of eight PAHs covered 3 or 4 orders of magnitude. The ranks of PNEC_chronic,10_s for eight PAHs are NAP > FLO > ACE > PHE > FLA > PYR > ANT > B[a]P. It seems that PAHs with higher molecular weights and more benzene rings have lower PNECs, which means higher sensitivity from aquatic organisms. The PNECs from HC_10_ and HC_5_ for one PAH are close. For B[a]P, FLA, and PHE, the acute PNECs based on HC_10_ or HC_5_ are larger than the acute PNECs based on HC_10_ or HC_5_; the ratios of the chronic PNECs based on the acute HC_10_ to those based on the chronic HC_10_ range from 0.13 to 0.77, indicating that the difference is small and the former are more protective. The peer-reviewed literature [24,27,28,29,31,40] and government documents [45,46,47,48] relevant to the water quality criteria (WQC) for PAHs are displayed in Table 5. In the relevant literature, the ECOTOX database is universally the primary source of data, and the data utilized include acute data (LC_50_ or EC_50_), ACR-transformed data, and chronic data (LOEC, EC_10_, LC_50_/3, and EC_50_/3). The logistic function is the most frequently employed fitting function, followed by the normal and burr type III functions. In general, the WQCs in the present study are lower compared to the relevant literature. The difference between WQCs from the government documents and this study is no more than one order of magnitude, the majority of which is no more than three times. It deserves attention that the difference between the WQCs from the literature using chronic or ACR-transformed data and the public documents is significant and ranges from one to four orders of magnitude, which may be a result of not only different toxicity data and derivation methods, but also the combination of freshwater and saltwater data used. The ratio of saltwater to freshwater HC_5_ for PAHs on microalgae was greater than 10 [14], indicating that freshwater species may be more sensitive to PAHs than saltwater species, and caution should be exercised when combing data from freshwater and saltwater. In conclusion, the AF_10_ calculated for PAHs is appropriate, and the derived PNECs are credible in the present study. The derivation method used for PNECs in this research improves the quality of the derived PNECs with long-term protection when lacking chronic toxicity data.

## 4. Conclusions

Based on the acute toxicity data for eight PAHs (ACE, ANT, B[a]P, FLA, FLO, NAP, PHE, and PYR) and the chronic toxicity data for four PAHs (B[a]P, FLA, FLO, and PHE), the optimal SSD models of each PAH were established using multiple nonlinear fitting functions. The key findings are as follows: (1) the ACR method is appropriate for calculating PNECs for PAHs; (2) the acute PNEC multiplied by the coefficient 0.1 can be used for the estimation of the chronic PNEC for PAHs lacking chronic toxicity data; (3) the AF_10_ used to calculate PNEC based on HC_10_ is 10 for PAHs; and (4) the chronic PNECs based on the acute HC_10_ and AF_10_ for eight PAHs are derived. This research provides practical ideas for deriving chronic PNECs for PAHs with insufficient chronic toxicity data.

## Figures and Tables

**Table 1:** The acute toxicity values (AVs) and/or chronic toxicity values (CVs) of eight polycyclic aromatic hydrocarbons (PAHs) on multiple species (μg/L).

**Rank**	**Acenaphthene(ACE)**		**Anthracene(ANT)**		**Benzo[a]pyrene(B[a]P)**				**Fluoranthene(FLA)**			
	**Species**	**AV**	**Species**	**AV**	**Species**	**AV**	**Species**	**CV**	**Species**	**AV**	**Species**	**CV**
1	*Paratanytarsus* sp.	60	*Aedes aegypti*	1	*Chlorella fusca var. vacuolata*	0.6308	*Zacco platypus*	0.2	*Lumbriculus variegatus*	1.2	*Crassostrea virginica*	10
2	*Daphnia magna*	120	*Lepomis macrochirus*	2.578	*Palaemonetes pugio*	1	*Daphnia magna*	0.3	*Hydra americana*	2.2	*Pimephales promelas*	10.4
3	*Tallaperla maria*	240	*Raphidocelis subcapitata*	3.3	*Daphnia magna*	1.298	*Carassius auratus*	0.3	*Oncorhynchus mykiss*	7.7	*Daphnia magna*	17
4	*Raphidocelis subcapitata*	322	*Lepomis* sp.	11.92	*Limnodrilus hoffmeisteri*	1.642	*Chanos chanos*	0.82	*Aedes aegypti*	10	*Chironomus tentans*	20
5	*Gammarus minus*	460	*Chlorella fusca var. vacuolata*	18.54	*Chironomus plumosus*	1.851	*Cyprinus flammans*	0.96	*Pimephales promelas*	12.2	*Chironomus riparius*	43
6	*Salmo trutta*	580	*Daphnia magna*	33.59	*Cyprinus flammans*	3.626	*Misgurnus anguillicaudatus*	8.681	*Physella virgata*	18.87	*Stylaria lacustris*	115
7	*Oncorhynchus mykiss*	670	*Culex quinquefasciatus*	37	*Scenedesmus acutus*	5	*Physella acuta*	10	*Lepomis macrochirus*	20.86	*Misgurnus anguillicaudatus*	269
8	*Lepomis macrochirus*	1700	*Aedes taeniorhynchus*	260	*Daphnia pulex*	5	*Eurytemora affinis*	12	*Gammarus minus*	32	*Hyalella azteca*	418.7
9	*Ictalurus punctatus*	1720	*Daphnia pulex*	754	*Rhodeus sinensis*	5			*Ictalurus punctatus*	36	*Pseudorasbora parva*	798
10	*Pimephales promelas*	1732	*Hyalella azteca*	873.70	*Chironomus riparius*	5			*Culex quinquefasciatus*	45	*Diporeia* sp.	861.6
11	*Paratanytarsus parthenogeneticus*	1800			*Rana limnocharis*	5.264			*Ceriodaphnia dubia*	45		
12	*Tanytarsus dissimilis*	2000			*Raphidocelis subcapitata*	6.9			*Aedes taeniorhynchus*	48		
13	*Aplexa hypnorum*	2040			*Macrobrachium nipponense*	7.632			*Xenopus laevis*	52		
14					*Misgurnus anguillicaudatus*	29.98			*Eohaustorius estuarus*	70		
15					*Eurytemora affinis*	58			*Gammarus pseudolimnaeus*	108		
16					*Danio rerio*	131.2			*Daphnia magna*	117		
17					*Xenopus laevis*	3331			*Homarus americanus*	120		
18					*Anabaena flosaquae*	4000			*Tallaperla maria*	135		
19					*Chlamydomonas reinhardtii*	4000			*Physa heterostropha*	137		
20					*Euglena gracilis*	4000			*Ophiogomphus* sp.	139.9		
21					*Poteriochromonas malhamensis*	4000			*Stylaria lacustris*	220		
22									*Chironomus tentans*	250		
23									*Lithobates pipiens*	276		
24									*Misgurnus anguillicaudatus,*	1887		
25									*Hydra* sp.	2032		
26									*Macrobrachium nipponense*	3011		
27									*Pseudorasbora parva*	5177		
28									*Rhodeus sinensis*	6251		
29									*Limnodrilus hofmeisteri*	6313		
30									*Chironomus plumosus*	7628		
31									*Rana limnocharis*	8695		
**Rank**	**Fluorene(FLO)**				**Naphthalene(NAP)**		**Phenanthrene(PHE)**				**Pyrene(PYR)**	
	**Species**	**AV**	**Species**	**CV**	**Species**	**AV**	**Species**	**AV**	**Species**	**CV**	**Species**	**AV**
1	*Daphnia pulex*	212	*Lepomis macrochirus*	125	*Melanotaenia fluviatilis*	213	*Coldwater Shrimp*	27	*Oncorhynchus mykiss*	5	*Daphnia magna*	6.579
2	*Daphnia magna*	430	*Daphnia magna*	125	*Micropterus salmoides*	240	*Lepomis macrochirus*	49	*Carassius auratus*	50	*Callinectes sapidus*	10
3	*Gammarus pseudolimnaeus*	600	*Chironomus riparius*	290	*Daphnia pulex*	1000	*Oncorhynchus mykiss*	50	*Daphnia pulex*	60	*Hyoplax formosensis*	11
4	*Lepomis macrochirus*	910	*Raphidocelis subcapitata*	3330	*Oncorhynchus mykiss*	1897.367	*Micropterus salmoides*	70	*Oryzias latipes*	100	*Neomysis awatschensis*	15
5	*Oncorhynchus mykiss*	1281	*Chara* sp.	35,000	*Macrobrachium kistnensis*	2000	*Hydra* sp.	96	*Daphnia magna*	191	*Chlorella fusca var. vacuolata*	25.71
6	*Chironomus plumosus*	2350			*Xenopus laevis*	2100	*Gammarus pseudolimnaeus*	126	*Rhodeus sinensis*	435	*Aedes aegypti*	35
7	*Chironomus riparius*	2350			*Callinectes sapidus*	2450	*Neomysis awatschensis*	126	*Misgurnus anguillicaudatus*	540	*Culex quinquefasciatus*	37
8	*Raphidocelis subcapitata*	3400			*Macrobrachium superbum*	2500	*Ptychocheilus lucius*	126	*Scenedesmus subspicatus*	2750	*Aedes taeniorhynchus*	60
9	*Pleuroceridae*	5600			*Chironomus tentans*	2810	*Eohaustorius estuarus*	158	*Scenedesmus armatus*	5000	*Pimephales promelas*	200
10	*Pimephales promelas*	100,000			*Oncorhynchus kisutch*	2986.212	*Pseudorasbora parva*	220			*Oncorhynchus mykiss*	2000
11					*Lepomis macrochirus*	3200	*Daphnia magna*	275			*Raphidocelis subcapitata*	894,000
12					*Daphnia magna*	3672.187	*Diporeia* sp.	295				
13					*Gammarus minus*	3930	*Raphidocelis subcapitata*	324				
14					*Physa gyrina*	5020	*Daphnia pulex*	350				
15					*Pimephales promelas*	5612.078	*Lumbriculus variegatus*	419				
16					*Oreochromis niloticus*	5900	*Gammarus minus*	460				
17					*Tilapia zillii*	5900	*Chironomus plumosus*	462				
18					*Raphidocelis subcapitata*	10,000	*Cyprinodon variegatus*	478				
19					*Lampetra tridentata*	10,000	*Oncorhynchus tshawytscha*	478				
20					*Tanytarsus dissimilis*	12,398.39	*Chironomus tentans*	490				
21					*Chironomus attenuatus*	13,000	*Hyalella azteca*	564.5				
22					*Scylla serrata*	17,700	*Oreochromis mossambicus*	600				
23					*Chlorella vulgaris*	33,000	*Rana limnocharis*	631				
24					*Diaptomus forbesi*	67,800	*Limnodrilus hoffmeisteri*	799				
25					*Gambusia affinis*	150,000	*Tanichthys albonubes*	913				
26							*Macrobrachium nipponense*	1079				
27							*Rhodeus sinensis*	2550				
28							*Lutjanus erythropterus*	3170				
29							*Misgurnus anguillicaudatus*	3684				

**Table 2:** The optimal fitting parameters (*a* and *b*) and goodness of fit (*R*^2^ and RMSE_50_) of species sensitivity distribution (SSD) models for the eight polycyclic aromatic hydrocarbons (PAHs).

PAH	Abbr.	SSD Data *	SSD Model	*a*	*b*	*R* ^2^	RMSE_50_
Acenaphthene	ACE	AV	Weibull	−5.92	1.92	0.9284	0.0105
Anthracene	ANT	AV	Dagum	0.45	3.20	0.9769	0.0377
Benzo[a]pyrene	B[a]P	AV	Gompertz	1.78	0.89	0.9301	0.0638
		CV	Gompertz	0.73	1.17	0.9240	0.0814
		CV_ES_	Gompertz	1.04	0.89	0.9300	0.0638
Fluoranthene	FLA	AV	Dagum	0.50	7.46	0.9786	0.0288
		CV	Gompertz	5.39	1.12	0.9563	0.0681
		CV_ES_	Dagum	0.52	3.95	0.9778	0.0280
Fluorene	FLO	AV	Error	1.10	3.51	0.9819	0.0103
		CV_ES_	Error	1.10	2.87	0.9819	0.0103
Naphthalene	NAP	AV	Dagum	0.98	2585.31	0.9872	0.0426
Phenanthrene	PHE	AV	Weibull	−6.40	2.36	0.9827	0.0300
		CV	Gudermannian	1.27	2.96	0.9818	0.0549
		CV_ES_	Weibull	−4.74	2.36	0.9827	0.0300
Pyrene	PYR	AV	Gompertz	6.83	1.49	0.9607	0.0448

* AV refers to the acute toxicity value, CV to the chronic toxicity value observed, and CV_ES_ to the chronic value estimated by the acute-to-chronic ratio (ACR) method.

**Table 3:** The acute-to-chronic ratios on various species (SACRs) and final acute-to-chronic ratios (FACRs) of B[a]P, FLA, FLO, and PHE.

PAH	Group	Species	AV * (μg/L)	CV * (μg/L)	SACR (AV/CV)	FACR
B[a]P	Crustaceans	*Daphnia magna*	1.298	0.3	4.33	4.06
	Crustaceans	*Eurytemora affinis*	58	12	4.83	
	Fish	*Misgurnus anguillicaudatus*	29.98	8.681	3.45	
	Fish	*Cyprinus flammans*	3.626	0.96	3.78	
FLA	Insect	*Chironomus tentans*	250	20	12.50	4.54
	Crustaceans	*Daphnia magna*	117	17	6.88	
	Fish	*Pimephales promelas*	12.2	10.4	1.17	
	Worms	*Stylaria lacustris*	220	115	1.91	
	Fish	*Pseudorasbora parva*	5177	798	6.49	
	Fish	*Misgurnus anguillicaudatus*	1887	269	7.01	
FLO	Insect	*Chironomus riparius*	2350	290	8.10	3.79
	Crustaceans	*Daphnia magna*	430	125	3.44	
	Fish	*Lepomis macrochirus*	910	125	7.28	
	Algae	*Raphidocelis subcapitata*	3400	3330	1.02	
PHE	Crustaceans	*Daphnia magna*	275	191	1.44	5.07
	Crustaceans	*Daphnia pulex*	350	60	5.83	
	Fish	*Misgurnus anguillicaudatus*	3684	540	6.82	
	Fish	*Oncorhynchus mykiss*	50	5	10.00	
	Fish	*Rhodeus sinensis*	2550	435	5.86	

* AV refers to the acute toxicity value, CV to the chronic toxicity value observed.

**Table 4:** Two hazardous concentrations (HC_5_ and HC_10_) and predicted no-effect concentrations (PNECs) for eight polycyclic aromatic hydrocarbons (PAHs) (μg/L) *.

PAH	Data **	HC_5_	HC_10_	PNEC_acute,5_ ***	PNEC_chronic,5_ ***	PNEC_acute,10_ ****	PNEC_chronic,10_ ****
ACE	AV	34.38	81.20	6.876	0.6876	8.120	0.8120
ANT	AV	0.3770	0.8925	0.07540	0.007540	0.08925	0.008925
B[a]P	AV	0.2629	0.5202	0.05258	0.005258	0.05202	0.005202
	CV	0.06205	0.1042		0.01241		0.01042
	CV_ES_	0.06472	0.1285		0.01294		0.01285
FLA	AV	4.087	7.602	0.8175	0.08175	0.7602	0.07602
	CV	3.333	5.704		0.6666		0.5704
	CV_ES_	0.7831	1.557		0.1566		0.1557
FLO	AV	135.8	232.8	27.16	2.716	23.28	2.328
	CV_ES_	35.83	61.23		7.166		6.123
NAP	AV	973.9	1275	194.8	19.48	127.5	12.75
PHE	AV	28.43	57.31	5.686	0.5686	5.731	0.5731
	CV	2.144	7.460		0.4287		0.7460
	CV_ES_	5.607	11.30		1.121		1.130
PYR	AV	3.572	5.360	0.7143	0.07143	0.5360	0.05360

* All PNECs retain four valid numbers. ** AV refers to the acute toxicity value, CV to the chronic toxicity value observed, and CV_ES_ to the chronic value estimated by the acute-to-chronic ratio (ACR) method. *** PNEC_acute,5_ = acute HC_5_/AF_5_; PNEC_chronic,5_ = acute HC_5_/AF_5_/10 or chronic HC_5_/AF_5_ (AF_5_ = 5). **** PNEC_acute,10_ = acute HC_10_/AF_10_; PNEC_chronic,10_ = acute HC_10_/AF_10_/10 or chronic HC_10_/AF_10_ (AF_10_ = 10).

**Table 5:** The water quality criteria (WQC) for eight polycyclic aromatic hydrocarbons (PAHs) from the peer-reviewed literature and public documents (μg/L).

Type	Source	ACE	ANT	B[a]P	FLA	FLO	NAP	PHE	PYR	Note ^a^
This study ^b^		0.8120	0.008925	0.005202	0.07602	2.328	12.75	0.5731	0.05360	LC_50_ or EC_50_; SSD
Peer-reviewed literature	[31] ^c^			2.33				1.09	0.011	NOEC; SSD
	[29]							112.3		LC_50_ or EC_50_; SSD
	[27] ^c^			11.408						LC_50_ or EC_50_; SSD
	[40] ^c^				6.25		61.6	5.17	5.28	EC_10_ or EC_50_/3 or LC_50_/3 or LOEC; SSD
	[24] ^c^			27.68		41.28				ACR-transformed LC_50_ or EC_50_; SSD
	[28]				174.6					ACR-transformed LC_50_ or EC_50_; SSD
Government document	[45] ^d^						16			SSD
	[46] ^e^	5.8		0.015	0.04	3	1.1	0.4	0.025	AF
	[47] ^f^			0.0028						—
	[48] ^g^			0.00017	0.0063		2			—

^a^ Data type and/or derivation method, in which SSD refers to species sensitivity distribution, and AF to assessment factor. ^b^ Only use freshwater data. ^c^ Use the combination of freshwater and saltwater data. ^d^ Trigger values for freshwater (95% protection level). ^e^ Water quality guidelines for the protection of aquatic life. ^f^ Concentration limit in centralized surface water sources of drinking water in China. ^g^ Annual average value of environmental quality standards for inland surface waters.

## Data Availability

Data is contained within the Supplementary Material.

## Supplementary Materials

The following supporting information can be downloaded at: https://www.mdpi.com/article/10.3390/toxics11070563/s1, Table S1: Nine two-parameter functions (y = f(a, b, x) used to construct the SSD models, Table S2: Acute toxicity values (AVs) (S_n_ = 13) of acenaphthene (ACE) to aquatic organisms, Table S3: Acute toxicity values (AVs) (S_n_ = 10) of anthracene (ANT) to aquatic organisms, Table S4: Acute toxicity values (AVs) (S_n_ = 21) and chronic toxicity values (CVs) (S_n,c_ = 8) of benzo[a]pyrene (B[a]P) to aquatic organisms, Table S5: Acute toxicity values (AVs) (S_n_ = 31) and chronic toxicity values (CVs) (S_n,c_ = 10) of fluoranthene (FLA) to aquatic organisms; Table S6: Acute toxicity values (AVs) (S_n_ = 10) and chronic toxicity values (CVs) (S_n,c_ = 5) of fluorene (FLO) to aquatic organisms, Table S7: Acute toxicity values (AVs) (S_n_ = 25) of naphthalene (NAP) to aquatic organisms, Table S8: Acute toxicity values (AVs) (S_n_ = 29) and chronic toxicity values (CVs) (S_n,c_ = 9) of phenanthrene (PHE) to aquatic organisms, Table S9: Acute toxicity values (AVs) (S_n_ = 11) of pyrene (PYR) to aquatic organisms, Table S10: Species groups involved in the toxicity data of the polycyclic aromatic hydrocarbons (PAHs), Table S11: The fitting parameters (*a* and *b*) and goodness of fit (*R*^2^, RMSE and RMSE_50_) of various SSD models based on the acute toxicity values (AVs) of the eight PAHs, Table S12: The fitting parameters (*a* and *b*) and goodness of fit (*R*^2^, RMSE and RMSE_50_) of various SSD models based on the chronic toxicity values (CVs) or ACR-transformed values (CV_ES_s) of the four PAHs, Table S13: The comparison between the SSD curves based on the acute toxicity value (AV), chronic toxicity value (CV) and ACR-transformed toxicity value (CVES) from three perspectives [28,49,50,51,52,53,54,55,56,57,58,59,60,61,62,63,64,65,66,67,68,69,70,71,72,73,74,75,76,77,78,79,80,81,82,83,84,85,86,87,88,89,90,91,92,93,94,95,96,97,98,99,100,101,102,103,104,105,106,107,108,109,110,111,112,113,114,115,116,117,118,119,120,121,122,123,124,125,126,127,128,129,130,131,132,133,134,135,136,137,138,139,140,141,142,143].
