# Supplementary material for: Optimized Derivation of Predicted No-Effect Concentrations (PNECs) for Eight Polycyclic Aromatic Hydrocarbons (PAHs) Using HC10 Based on Acute Toxicity Data
Source: Toxics. 2023 Jun 28;11(7):563. doi: 10.3390/toxics11070563 (PMC10384761; doi:10.3390/toxics11070563)
Supplement: Supplementary file 1 [file toxics-11-00563-s001.zip › toxics-2459720-supplementary.pdf]

toxics

Supplementary Materials

Optimized Derivation of Predicted No-Effect Concentrations (PNECs) for Eight Polycyclic Aromatic Hydrocarbons (PAHs) Using HC_{10} Based on Acute Toxicity Data

Xiao Sun [1,2,3], Ting-Ting Ding [1,2], Ze-Jun Wang [4], Peng Huang [1,2] and Shu-Shen Liu [1,2,3,*]

1 Key Laboratory of Yangtze River Water Environment, Ministry of Education, College of Environmental Science and Engineering, Tongji University, Shanghai 200092, China
2 State Key Laboratory of Pollution Control and Resource Reuse, College of Environmental Science and Engineering, Tongji University, Shanghai 200092, China
3 Shanghai Institute of Pollution Control and Ecological Security, Shanghai 200092, China
4 National and Local Joint Engineering Laboratory of Municipal Sewage Resource Utilization Technology, School of Environmental Science and Engineering, Suzhou University of Science and Technology, Suzhou 215009, China
* Correspondence: ssliuhl@263.net; Tel.: +86-021-65982767

Table S1. Nine two-parameter functions (y=f(a, b, x) used to construct the SSD models.

Function	Expression
Arctangent	$y=\frac{\frac{\pi}{2}+\arctan(\frac{x-a}{b})}{\pi}$
Dagum	$y=\frac{1}{\left(1+10^{-ax}\right)^{b}}$
Error	$y=\frac{1+erf\left(ax-b\right)}{2}$
Gompertz	$y=e^{-ae^{-bx}}$
Gudermannian	$y=\frac{\frac{\pi}{2}+\arcsin\left(\tanh\left(ax-b\right)\right)}{\pi}$
Hyperbolic tangent	$y=\frac{1+\tanh\left(ax-b\right)}{2}$
Logistic	$y=\frac{1}{1+e^{\frac{-(x-a)}{b}}}$
Normal	$y=\frac{1}{b\sqrt{2\pi}}\int_{-\infty}^{x}e^{\frac{-(t-a)^2}{2b^2}}dt$
Weibull	$y=1-e^{-e^{a+bx}}$

Table S2. Acute toxicity values (AVs) (S_n=13) of acenaphthene (ACE) to aquatic organisms.

Aquatic biota	Species	Phylum	Family	Exposure Type	Duration (Days)	Endpoint	Effect	AV (μg/L)	Geom.* (μg/L)	Reference
Algae	*Raphidocelis subcapitata*	Chlorophyta	Chlorellaceae	Static	4	EC_{50}	Mortality	520		[49]
Crustaceans	*Daphnia magna*	Arthropoda	Daphniidae	Flow-through	2	LC_{50}	Mortality	120		[50]
Crustaceans	*Gammarus minus*	Arthropoda	Gammaridae	Static	4	LC_{50}	Growth rate	460		[51]
Fish	*Ictalurus punctatus*	Chordata	Ictaluridae	Flow-through	4	LC_{50}	Mortality	1720		[52]
Fish	*Lepomis macrochirus*	Chordata	Centrarchidae	Static	4	LC_{50}	Mortality	1700		[53]
Fish	*Oncorhynchus mykiss*	Chordata	Salmonidae	Flow-through	4	LC_{50}	Mortality	670		[52]
Fish	*Pimephales promelas*	Chordata	Cyprinidae	Flow-through	4	LC_{50}	Mortality	1400	1732	[50]
				Flow-through	4	LC_{50}	Mortality	1600		[52]
				Flow-through	4	LC_{50}	Mortality	1730		[54]
				Flow-through	4	LC_{50}	Mortality	608		[55]
				Static	4	LC_{50}	Mortality	3700		[56]
				Static	4	LC_{50}	Mortality	3100		[57]
Fish	*Salmo trutta*	Chordata	Salmonidae	Static	4	LC_{50}	Mortality	580		[52]
Insects	*Paratanytarsus parthenogeneticus*	Arthropoda	Chironomidae	Static	2	LC_{50}	Mortality	1800		[58]

Insects	*Paratanytarsus sp.*	Arthropoda	Chironomidae	Static	2	LC_{50}	Mortality	60		[59]
Insects	*Tanytarsus dissimilis*	Arthropoda	Chironomidae	Static	2	LC_{50}	Mortality	2000		[60]
Insects	*Tallaperla maria*	Arthropoda	Peltoperlidae	Static	4	LC_{50}	Mortality	240		[51]
Molluscs	*Aplexa hypnorum*	Mollusca	Physidae	Flow-through	4	LC_{50}	Mortality	2040		[52]

* Geom. refers to the geometrical mean of several values for one Species under one endpoint.

Table S3. Acute toxicity values (AVs) (S_n=10) of anthracene (ANT) to aquatic organisms.

**Aquatic biota**	**Species**	**Phylum**	**Family**	**Exposure Type**	**Duration (Days)**	**Endpoint**	**Effect**	**AV (μg/L)**	**Geom.* (μg/L)**	**Reference**
Algae	*Chlorella fusca var. vacuolata*	Chlorophyta	Chlorellaceae	Static	1	EC_{50}	Growth rate	18.54		[61]
Algae	*Raphidocelis subcapitata*	Chlorophyta	Chlorellaceae	Renewal	1	EC_{50}	Physiology	3.3		[62]
Crustaceans	*Daphnia magna*	Arthropoda	Daphniidae	Static	2	EC_{50}	Immobilization	35.65	33.59	[63]
				Static	2	EC_{50}	Immobilization	95		[64]
					2	EC_{50}	Survival	11.19		[65]
Crustaceans	*Daphnia pulex*	Arthropoda	Daphniidae	Static	2	EC_{50}	Immobilization	754		[66]
Crustaceans	*Hyalella azteca*	Arthropoda	Gammaridae	Renewal	4	EC_{50}	Survival	873.7		[67]
Fish	*Lepomis macrochirus*	Chordata	Centrarchidae	Flow-through	4	LC_{50}	Mortality	4.5	2.578	[68]
				Flow-through	4	LC_{50}	Mortality	2.78		[69]

				Flow-through	4	LC_{50}	Mortality	1.27		[70]
				Static	4	LC_{50}	Mortality	2.78		[71]
Fish	*Lepomis sp.*	Chordata	Centrarchidae	Flow-through	4	LC_{50}	Mortality	11.92		[69]
Insect	*Aedes aegypti*	Arthropoda	Culicidae	Static	1	LC_{50}	Mortality	1		[72]
Insect	*Aedes taeniorhynchus*	Arthropoda	Culicidae	Static	1	LC_{50}	Mortality	260		[72]
Insect	*Culex quinquefasciatus*	Arthropoda	Culicidae	Static	1	LC_{50}	Mortality	37		[72]

* Geom. refers to the geometrical mean of several values for one Species under one endpoint.

Table S4. Acute toxicity values (AVs) (S_n=21) and chronic toxicity values (CVs) ($S_{n,c}$=8) of benzo[a]pyrene (B[a]P) to aquatic organisms.

**Aquatic biota**	**Species**	**Phylum**	**Family**	**Exposure Type**	**Duration (Days)**	**Endpoint**	**Effect**	**AV/CV (μg/L)**	**Reference**
Algae	*Anabaena flosaquae*	Cyanophyta	Nostocaceae	Static	3	EC_{50}	Growth	4000	[73]
Algae	*Chlamydomonas reinhardtii*	Chlorophyta	Chlamydomonadaceae	Static	3	EC_{50}	Growth	4000	[73]
Algae	*Chlorella fusca var. vacuolata*	Chlorophyta	Chlorellaceae	Static	1	EC_{50}	Growth rate	0.6308	[71]
Algae	*Euglena gracilis*	Euglenozoa	Euglenaceae	Static	3	EC_{50}	Growth	4000	[74]
Algae	*Poteriochromonas malhamensis*	Chrysophyta	Ochromonadaceae	Static	3	EC_{50}	Growth	4000	[73]
Algae	*Raphidocelis subcapitata*	Chlorophyta	Chlorellaceae	Static	2	EC_{50}	Growth rate	6.9	[74]
Algae	*Scenedesmus acutus*	Chlorophyta	Scenedesmaceae	Static	3	EC_{50}	Growth	5	[73]
Amphibians	*Rana limnocharis*	Chordata	Ranidae	Renewal	4	LC_{50}	Mortality	5.264	[28]

Amphibians	*Xenopus laevis*	Chordata	Pipidae	Renewal	4	EC_{50}	Development	3331	[75]
Annelida	*Limnodrilus hoffmeisteri*	Annelida	Tubificidae	Renewal	4	LC_{50}	Mortality	1.642	[28]
Crustaceans	*Daphnia magna*	Arthropoda	Daphniidae		2	LC_{50}	Mortality	1.298	[76]
Crustaceans	*Daphnia pulex*	Arthropoda	Daphniidae	Static	4	LC_{50}	Mortality	5	[77]
Crustaceans	*Eurytemora affinis*	Arthropoda	Daphniidae		4	LC_{50}	Mortality	58	[78]
Crustaceans	*Macrobrachium nipponense*	Arthropoda	Palaemonidae	Renewal	4	LC_{50}	Mortality	7.632	[28]
Crustaceans	*Palaemonetes pugio*	Arthropoda	Palaemonidae		4	LC_{50}	Mortality	1	[78]
Fish	*Cyprinus flammans*	Chordata	Cyprinidae	Renewal	4	LC_{50}	Mortality	3.626	[76]
Fish	*Danio rerio*	Chordata	Cyprinidae	Static	3	EC_{50}	Development	131.2	[79]
Fish	*Misgurnusanguillicaudatus*	Chordata	Cobitidae	Renewal	4	LC_{50}	Mortality	29.98	[76]
Fish	*Rhodeus sinensis*	Chordata	Cyprinidae	Renewal	4	LC_{50}	Mortality	5	[28]
Insect	*Chironomus plumosus*	Arthropoda	Chironomidae	Renewal	4	LC_{50}	Mortality	1.851	[28]
Insect	*Chironomus riparius*	Arthropoda	Chironomidae	Static	1	EC_{50}	Intoxication	5	[80]
Crustaceans	*Daphnia magna*	Arthropoda	Daphniidae	Static-renewal	21	LC10	Mortality	0.3	[76]
Crustaceans	*Eurytemora affinis*	Arthropoda	Daphniidae	Renewal	21	NOEC	Development	12	[81]
Fish	*Carassius auratus*	Chordata	Cyprinidae	Renewal	12	NOEC	Enzyme	0.3	[82]
Fish	*Chanos chanos*	Chordata	Chanidae	Chanidae	28	NOEC	growth	0.82	[83]
Fish	*Cyprinus flammans*	Chordata	Cyprinidae	Static-renewal	28	LC10	Mortality	0.96	[76]
Fish	*Misgurnusanguillicaudatus*	Chordata	Cobitidae	Static-renewal	28	LC10	Mortality	8.681	[76]

Fish	*Zacco platypus*	Chordata	Cyprinidae	Flow-through	14	NOEC	Enzyme	0.2	[84]
Molluscs	*Physella acuta*	Mollusca	Physidae	Renewal	21	NOEC	Mortality	10	[85]

Table S5. Acute toxicity values (AVs) (S_n=31) and chronic toxicity values (CVs) ($S_{n,c}$=10) of fluoranthene (FLA) to aquatic organisms.

Aquatic biota	Species	Phylum	Family	Exposure Type	Duration (Days)	Endpoint	Effect	AV/CV (μg/L)	Geom.* (μg/L)	Reference
Amphibians	*Lithobates pipiens*	Chordata	Ranidae	Renewal	4	EC_{50}	Development	276		[28]
Amphibians	*Rana limnocharis*	Chordata	Ranidae	Renewal	4	LC_{50}	Mortality	8695		[28]
Amphibians	*Xenopus laevis*	Chordata	Pipidae	Renewal	4	EC_{50}	Development	52		[28]
Crustaceans	*Macrobrachium nipponense*	Arthropoda	Palaemonidae	Renewal	4	LC_{50}	Mortality	3011		[86]
Crustaceans	*Ceriodaphnia dubia*	Arthropoda	Daphniidae	Static	2	LC_{50}	Mortality	45		[87]
Crustaceans	*Daphnia magna*	Arthropoda	Daphniidae	Renewal	2	EC_{50}	Mortality	117		[88]
Crustaceans	*Eohaustorius estuarus*	Arthropoda	Gammaridae		4	LC_{50}	Mortality	70		[78]
Crustaceans	*Gammarus minus*	Arthropoda	Gammaridae	Static	4	LC_{50}	Mortality	32		[89]
Crustaceans	*Gammarus pseudolimnaeus*	Arthropoda	Gammaridae	Flow-through	4	LC_{50}	Mortality	108		[90]
Crustaceans	*Homarus americanus*	Arthropoda	Nephropidae		4	LC_{50}	Mortality	120		[78]
Fish	*Ictalurus punctatus*	Chordata	Ictaluridae	Static	4	LC_{50}	Mortality	36		[91]
Fish	*Lepomis macrochirus*	Chordata	Centrarchidae	Flow-through		EC_{50}	Behavior	43.5	20.86	[88]
				Flow-through		LC_{50}	Mortality	10		[90]

Aquatic biota	Species	Phylum	Family	Exposure Type	Duration (Days)	Endpoint	Effect	AV/CV (µg/L)	Geom.* (µg/L)	Reference
Fish	*Misgurnus anguillicaudatus*	Chordata	Cobitidae	Renewal	4	LC_{50}	Mortality	1887		[28]
Fish	*Oncorhynchus mykiss*	Chordata	Salmonidae	Flow-through	4	LC_{50}	Mortality	7.7		[92]
Fish	*Pimephales promelas*	Chordata	Cyprinidae	Flow-through	4	LC_{50}	Mortality	12.2		[92]
Fish	*Pseudorasbora parva*	Chordata	Cyprinidae	Renewal	4	LC_{50}	Mortality	5177		[28]
Fish	*Rhodeus sinensis*	Chordata	Cyprinidae	Renewal	4	LC_{50}	Mortality	6251		[28]
Invertebrates	*Hydra americana*	Cnidaria	Hydridae	Flow-through	4	LC_{50}	Mortality	2.2		[90]
Insect	*Aedes aegypti*	Arthropoda	Culicidae	Static	1	LC_{50}	Mortality	10		[72]
Insect	*Aedes taeniorhynchus*	Arthropoda	Culicidae	Static	1	LC_{50}	Mortality	48		[72]
Insect	*Chironomus plumosus*	Arthropoda	Chironomidae	Renewal	4	LC_{50}	Mortality	7628		[28]
Insect	*Culex quinquefasciatus*	Arthropoda	Culicidae	Static	1	LC_{50}	Mortality	45		[72]
Insect	*Chironomus tentans*	Arthropoda	Chironomidae	Static	2	EC_{50}	Behavior	250		[92]
Insect	*Ophiogomphus sp.*	Arthropoda	Gomphidae	Flow-through		LC_{50}	Mortality	110	139.9	[90]
				Flow-through		LC_{50}	Mortality	178		[88]
Insect	*Tallaperla maria*	Arthropoda	Peltoperlidae	Static	4	LC_{50}	Mortality	135		[89]
Molluscs	*Physa heterostropha*	Mollusca	Retusidae	Static	4	LC_{50}	Mortality	137		[89]

Molluscs	*Physella virgata*	Mollusca	Retusidae	Flow-through		LC_{50}	Mortality	178	18.87	[88]
				Flow-through		LC_{50}	Mortality	2		[90]
Worms	*Lumbriculus variegatus*	Annelida	Lumbriculidae	Flow-through	4	LC_{50}	Mortality	1.2		[90]
Worms	*Stylaria lacustris*	Annelida	Naididae	Static	2	LC_{50}	Mortality	220		[93]
Invertebrates	*Hydra sp.*	Coelenterata	Hydridae	Renewal	4	LC_{50}	Mortality	2032		[28]
Invertebrates	*Limnodrilus hofmeisteri*	Annelida	Tubificidae	Renewal	4	LC_{50}	Mortality	6313		[28]
Crustaceans	*Daphnia magna*	Daphniidae	Arthropoda	Renewal	21	NOEC	Reproduction	17		[88]
Crustaceans	*Diporeia sp.*	Gammaridae	Arthropoda	Leaching	16	NOEC	Mortality	861.6		[94]
Crustaceans	*Hyalella azteca*	Gammaridae	Arthropoda	Leaching	30	NOEC	Mortality	418.7		[94]
Fish	*Pimephales promelas*	Cyprinidae	Flow-through	Chordata	32	NOEC	Mortality	10.4		[90]
Fish	*Pseudorasbora parva*	Chordata	Cyprinidae	Renewal	21	EC_{10}	Longevity	798		[28]
Fish	*Misgurnus anguillicaudatus*	Chordata	Cobitidae	Renewal	21	EC_{10}	Longevity	269		[28]
Insect	*Chironomus riparius*	Chironomidae	Arthropoda	Leaching	17.6	NOEC	Development	43		[95]
Insect	*Chironomus tentans*	Chironomidae	Arthropoda	Static	10	NOEC	Mortality	20		[93]
Molluscs	*Crassostrea virginica*	Ostreidae	Mollusca	Renewal	21	NOEC	Morphology	10		[96]
Worms	*Stylaria lacustris*	Naididae	Annelida	Static	10	NOEC	Mortality	115		[93]

* Geom. refers to the geometrical mean of several values for one Species under one endpoint.

Table S6. Acute toxicity values (AVs) (S_{n}=10) and chronic toxicity values (CVs) ($S_{n,c}$=5) of fluorene (FLO) to aquatic organisms.

Aquatic biota	Species	Phylum	Family	Exposure Type	Duration (Days)	Endpoint	Effect	AV/CV (μg/L)	Geom.* (μg/L)	Reference
Algae	*Raphidocelis subcapitata*	Chlorophyta	Chlorellaceae	Static	4	EC_{50}	Growth rate	3400		[97]
Crustaceans	*Daphnia magna*	Arthropoda	Daphniidae	Static	2	EC_{50}	Intoxication	430		[97]
Crustaceans	*Daphnia pulex*	Arthropoda	Daphniidae	Static	2	EC_{50}	Intoxication	212		[66]
Crustaceans	*Gammarus pseudolimnaeus*	Arthropoda	Gammaridae	Static	4	LC_{50}	Mortality	600		[97]
Fish	*Lepomis macrochirus*	Chordata	Centrarchidae	Static	4	LC_{50}	Mortality	910		[97]
Fish	*Oncorhynchus mykiss*	Chordata	Salmonidae	Static	4	LC_{50}	Mortality	820	1281	[97]
				Renewal	4	LC_{50}	Mortality	2000		[97]
Fish	*Pimephales promelas*	Chordata	Cyprinidae	Static	4	LC_{50}	Mortality	100000		[97]
Insects	*Chironomus plumosus*	Arthropoda	Chironomidae	Static	2	EC_{50}	Intoxication	2350		[98]
Insects	*Chironomus riparius*	Arthropoda	Chironomidae	Static	2	EC_{50}	Intoxication	2350		[97]
Molluscs	*Pleuroceridae*	Mollusca	Pleuroceridae	Static	4	LC_{50}	Mortality	5600		[97]
Algae	*Chara sp.*	Charophyta	Characeae	Static	21	NOEC	Growth	35000		[97]
Algae	*Raphidocelis subcapitata*	Chlorophyta	Chlorellaceae	Static	14	NOEC	Growth rate	3330		[97]
Crustaceans	*Daphnia magna*	Arthropoda	Daphniidae	Flow-through	21	NOEC	Reproduction	125		[97]
Fish	*Lepomis macrochirus*	Chordata	Centrarchidae	Flow-through	30	NOEC	Accumulation	125		[97]

Insect	*Chironomus riparius*	Arthropoda	Chironomidae	Flow-through	30	NOEC	Development	290	[97]

* Geom. refers to the geometrical mean of several values for one Species under one endpoint.

Table S7. Acute toxicity values (AVs) (S_n=25) of naphthalene (NAP) to aquatic organisms.

**Aquatic biota**	**Species**	**Phylum**	**Family**	**Exposure Type**	**Duration (Days)**	**Endpoint**	**Effect**	**AV/CV (μg/L)**	**Geom.* (μg/L)**	**Reference**
Algea	*Chlorella vulgaris*	Chlorophyta	Chlorellaceae	Static	1	EC_{50}	Growth	33000		[99]
Algea	*Raphidocelis subcapitata*	Chlorophyta	Chlorellaceae	Static	2	EC_{50}	Growth rate	10000		[74]
Amphibians	*Xenopus laevis*	Chordata	Pipidae	Flow-through	4	LC_{50}	Mortality	2100		[100]
Crustaceans	*Callinectes sapidus*	Arthropoda	Portunidae		4	LC_{50}		2450		[78]
Crustaceans	*Daphnia magna*	Arthropoda	Daphniidae	Static	2	EC_{50}	Intoxication	1600	3672	[101]
				Static	2	LC_{50}	Mortality	8600		[102]
				Static	2	EC_{50}	Intoxication	2194		[64]
				Static	2	EC_{50}	Intoxication	1600		[103]
				Static	2	LC_{50}	Mortality	22600		[104]
				Static	2	LC_{50}	Mortality	3400		[105]

				Static	2	LC_{50}	Mortality	2160	[106]
				Static	2	EC_{50}	Intoxication	4730	[63]
				Flow-through	2	EC_{50}	Mortality	1960	[107]
				Static	2	LC_{50}	Mortality	6000	[108]
Crustaceans	*Daphnia pulex*	Arthropoda	Daphniidae	Static	4	LC_{50}	Mortality	1000	[77]
Crustaceans	*Diaptomus forbesi*	Arthropoda	Diaptomidae	Static	4	LC_{50}	Mortality	67800	[109]
Crustaceans	*Gammarus minus*	Arthropoda	Gammaridae	Static	2	LC_{50}	Mortality	3930	[106]
Crustaceans	*Macrobrachium kistnensis*	Arthropoda	Palaemonidae	Renewal	4	LC_{50}	Mortality	2000	[110]
Crustaceans	*Macrobrachium superbum*	Arthropoda	Palaemonidae		4	LC_{50}		2500	[78]
Crustaceans	*Scylla serrata*	Arthropoda	Portunidae		4	LC_{50}		17700	[7]
Fish	*Gambusia affinis*	Chordata	Poeciliidae	Static	4	LC_{50}	Mortality	150000	[111]
Fish	*Lampetra tridentata*	Chordata	Petromyzonidae	Renewal	4	LC_{50}	Mortality	10000	[112]
Fish	*Lepomis macrochirus*	Chordata	Centrarchidae	Renewal	4	LC_{50}	Mortality	3200	[101]
Fish	*Melanotaenia fluviatilis*	Chordata	Melanotaeniidae		4	LC_{50}	Mortality	213	[113]
Fish	*Micropterus salmoides*	Chordata	Centrarchidae	Flow-through	3	LC_{50}	Mortality	240	[114]

Fish	*Oncorhynchus kisutch*	Chordata	Salmonidae	Flow-through	4	LC_{50}	Mortality	2100	2986	[101]
				Renewal	4	LC_{50}	Mortality	5600		[115]
				Flow-through	4	LC_{50}	Mortality	2100		[116]
				Flow-through	4	LC_{50}	Mortality	3220		[117]
Fish	*Oncorhynchus mykiss*	Chordata	Salmonidae	Static	4	LC_{50}	Mortality	1800	1897	[118]
				Renewal	4	LC_{50}	Mortality	2000		[101]
				Flow-through	4	LC_{50}	Mortality	1600		[119]
				Flow-through	4	LC_{50}	Mortality	2250		[120]
Fish	*Oreochromis niloticus*	Chordata	Cichlidae		4	LC_{50}		5900		[78]
Fish	*Pimephales promelas*	Chordata	Cyprinidae	Flow-through	4	LC_{50}	Mortality	6080	5612	[127]
				Static	4	LC_{50}	Mortality	1990		[106]
				Flow-through	4	LC_{50}	Mortality	6140		[121]
				Flow-through	4	LC_{50}	Mortality	6140		[122]
				Flow-through	4	LC_{50}	Mortality	7900		[119]
				Flow-through	4	LC_{50}	Mortality	4900		[120]
				Flow-through	4	LC_{50}	Mortality	9930		[107]
Fish	*Tilapia zillii*	Chordata	Cichlidae		4	LC_{50}	Mortality	5900		[122]

Insects	*Chironomus attenuatus*	Arthropoda	Chironomidae	Static	1	LC_{50}	Mortality	13000		[123]
Insects	*Chironomus tentans*	Arthropoda	Chironomidae	Static	2	LC_{50}	Mortality	2810		[106]
Insects	*Tanytarsus dissimilis*	Arthropoda	Chironomidae	Static	2	LC_{50}	Mortality	12600	12398	[123]
				Static	2	LC_{50}	Mortality	12200		[124]
Molluscs	*Physa gyrina*	Mollusca	Physidae	Static	2	LC_{50}	Mortality	5020		[106]

* Geom. refers to the geometrical mean of several values for one Species under one endpoint.

Table S8. Acute toxicity values (AVs) (S_n=29) and chronic toxicity values (CVs) ($S_{n,c}$=9) of phenanthrene (PHE) to aquatic organisms.

Aquatic biota	Species	Phylum	Family	Exposure Type	Duration (Days)	Endpoint	Effect	AV/CV (μg/L)	Reference
Algea	*Raphidocelis subcapitata*	Chlorophyta	Chlorellaceae	Static	3	EC_{50}	Growth rate	324	[125]
Amphibian	*Rana limnocharis*	Chordata	Ranidae	Static-renewal	4	LC_{50}	Mortality	631	[125]
Crustaceans	*Coldwater Shrimp*	Arthropoda	Pandalidae	Static-renewal	4	LC_{50}	Mortality	27	[125]
Crustaceans	*Daphnia pulex*	Arthropoda	Daphniidae	Static	4	LC_{50}	Intoxication	350	[66]
Crustaceans	*Daphnia magna*	Arthropoda	Daphniidae	Static-renewal	4	LC_{50}	Mortality	275	[125]
Crustaceans	*Diporeia sp.*	Arthropoda	Gammaridae	Renewal	2	EC_{50}	Intoxication	295	[128]
Crustaceans	*Eohaustorius estuarus*	Arthropoda	Gammaridae		4	LC_{50}	Mortality	158	[78]
Crustaceans	*Gammarus minus*	Arthropoda	Gammaridae	Static	2	LC_{50}	Mortality	460	[106]

Crustaceans	*Gammarus pseudolimnaeus*	Arthropoda	Gammaridae	Flow-through	4	EC_{50}	Intoxication	126	[129]
Crustaceans	*Hyalella azteca*	Arthropoda	Gammaridae	Renewal	4	EC_{50}	Mortality	564.5	[67]
Crustaceans	*Macrobrachium nipponense*	Arthropoda	Palaemonidae	Static-renewal	4	LC_{50}	Mortality	1079	[126]
Crustaceans	*Neomysis awatschensis*	Arthropoda	Mysidae	Renewal	4	LC_{50}	Mortality	126	[130]
Fish	*Cyprinodon variegatus*	Chordata	Cyprinodontidae		4	LC_{50}	Mortality	478	[78]
Fish	*Lepomis macrochirus*	Chordata	Centrarchidae	Flow-through	4	EC_{50}	Behavior	49	[129]
Fish	*Lutjanus erythropterus*	Chordata	Lutjanidae	semi-static	4	LC_{50}	Mortality	3170	[131]
Fish	*Micropterus salmoides*	Chordata	Centrarchidae	Flow-through	3	LC_{50}	Mortality	70	[114]
Fish	*Misgurnus anguillicaudatus*	Chordata	Cobitidae	Static-renewal	4	LC_{50}	Mortality	3684	[126]
Fish	*Oncorhynchus mykiss*	Chordata	Salmonidae	Flow-through	4	EC_{50}	Behavior	50	[129]
Fish	*Oncorhynchus tshawytscha*	Chordata	Salmonidae	Static-renewal	4	LC_{50}	Mortality	478	[126]
Fish	*Oreochromis mossambicus*	Chordata	Cichlidae	Static-renewal	4	LC_{50}	Mortality	600	[126]
Fish	*Pseudorasbora parva*	Chordata	Cyprinidae		4	LC_{50}	Mortality	220	[132]
Fish	*Ptychocheilus lucius*	Chordata	Cyprinidae	Static-renewal	4	LC_{50}	Mortality	126	[126]
Fish	*Rhodeus sinensis*	Chordata	Cyprinidae	Static-renewal	4	LC_{50}	Mortality	2550	[126]
Fish	*Tanichthys albonubes*	Chordata	Cyprinidae	Static	4	LC_{50}	Mortality	913	[133]

Table S8. (*Continued*).

Aquatic biota	Species	Phylum	Family	Exposure Type	Duration (Days)	Endpoint	Effect	AV/CV (μg/L)	Reference
Insects	*Chironomus plumosus*	Arthropoda	Chironomidae	Static-renewal	4	LC_{50}	Mortality	462	[126]
Insects	*Chironomus tentans*	Arthropoda	Chironomidae	Static	2	LC_{50}	Mortality	490	[106]
Invertebrates	*Hydra sp.*	Coelenterata	Hydridae	Flow-through	4	EC_{50}	Growth	96	[129]
Worms	*Lumbriculus variegatus*	Annelida	Lumbriculidae	Flow-through	4	EC_{50}	Intoxication	419	[129]
Worms	*Limnodrilus hoffmeisteri*	Annelida	Tubificidae	Static-renewal	4	LC_{50}	Mortality	799	[126]
Algae	*Scenedesmus armatus*	Chlorophyta	Scenedesmaceae	Static	7	NOEC	Growth rate	5000	[134]
Algae	*Scenedesmus subspicatus*	Chlorophyta	Scenedesmaceae	Static	7	NOEC	Growth rate	2750	[135]
Crustaceans	*Daphnia magna*	Arthropoda	Daphniidae	Static-renewal	21	EC_{10}	Growth	191	[126]
Crustaceans	*Daphnia pulex*	Arthropoda	Daphniidae	Renewal	16	NOEC	Growth	60	[136]
Fish	*Carassius auratus*	Chordata	Cyprinidae	Renewal	21	NOEC	Enzyme	50	[137]
Fish	*Misgurnus anguillicaudatus*	Chordata	Cobitidae	Static-renewal	28	EC_{10}	Growth	540	[12]
Fish	*Oncorhynchus mykiss*	Chordata	Salmonidae	Flow-through	90	NOEC	Mortality	5	[129]
Fish	*Oryzias latipes*	Chordata	Adrianichthyidae	Renewal	18	NOEC	Development	100	[138]
Fish	*Rhodeus sinensis*	Chordata	Cyprinidae	Static-renewal	28	EC_{10}	Growth	435	[126]

Table S9. Acute toxicity values (AVs) (S_n=11) of pyrene (PYR) to aquatic organisms.

Aquatic biota	Species	Phylum	Family	Exposure Type	Duration (Days)	Endpoint	Effect	AV/CV (μg/L)	Geom.* (μg/L)	Reference
Algae	*Chlorella fusca var. vacuolata*	Chlorophyta	Chlorellaceae	Static	1	EC_{50}	Growth rate	49.73	25.71	[139]
				Static	1	EC_{50}	Growth rate	48.54		[140]
				Static	1	EC_{50}	Growth rate	7.038		[61]
Algae	*Raphidocelis subcapitata*	Chlorophyta	Chlorellaceae	Static	3	EC_{50}	Intoxication	894000		[141]
Crustaceans	*Callinectes sapidus*	Arthropoda	Portunidae		4	LC_{50}	Mortality	10		[78]
Crustaceans	*Daphnia magna*	Arthropoda	Daphniidae	Static	1	EC_{50}	Mortality	10	6.579	[142]
					2	EC_{50}	Mortality	4.328		[65]
Crustaceans	*Hyoplax formosensis*	Arthropoda	Ocypodidae		4	LC_{50}	Mortality	11		[78]
Crustaceans	*Neomysis awatschensis*	Arthropoda	Mysidae	Renewal	4	LC_{50}	Mortality	15		[130]
Fish	*Oncorhynchus mykiss*	Chordata	Salmonidae	Renewal	4	LC_{50}	Mortality	2000		[143]
Fish	*Pimephales promelas*	Chordata	Cyprinidae		4	LC_{50}	Mortality	200		[78]
Insect	*Aedes aegypti*	Arthropoda	Culicidae	Static	1	LC_{50}	Mortality	35		[72]
Insect	*Aedes taeniorhynchus*	Arthropoda	Culicidae	Static	1	LC_{50}	Mortality	60		[72]
Insect	*Culex quinquefasciatus*	Arthropoda	Culicidae	Static	1	LC_{50}	Mortality	37		[72]

* Geom. refers to the geometrical mean of several values for one Species under one endpoint.

Table S10. Species groups involved in the toxicity data of the polycyclic aromatic hydrocarbons (PAHs).

PAH	n_{AV}*	n_{CV}*	Algae	Amphibian	Crustaceans	Fish	Insect	Invertebrate	Mollusca	Plant	Worm
ACE	13		√		√	√	√		√		
ANT	10		√		√	√	√				
B[a]P	21		√	√	√	√	√				
		8			√	√			√		
FLA	31			√	√	√	√	√	√		√
		10			√	√	√		√		√
FLO	10		√		√	√	√		√		
		5	√		√	√	√				
NAP	25		√	√	√	√	√		√		
PHE	29		√	√	√	√	√	√			√
		9	√		√	√					
PYR	11		√		√	√	√				

* n_{AV} and n_{CV} refer to the number of samples having acute and chronic toxicity values, respectively.

Table S11. The fitting parameters (*a* and *b*) and goodness of fit (R^2, RMSE and $RMSE_{50}$) of various SSD models based on the acute toxicity values (AVs) of the eight PAHs.

PAH	SSD model	*a*	*b*	R^2	$RMSE_{50}$	RMSE
ACE	Arctangent	2.86	0.40	0.9146	0.02243	0.08313
	Dagum	0.80	128.66	0.9131	0.05594	0.08843
	Error	1.15	3.29	0.9243	0.02826	0.08089
	Gompertz	127.31	1.85	0.9131	0.05643	0.08854
	Gudermannian	2.16	6.18	0.9236	0.01617	0.08043
	Hyperbolic tangent	1.34	3.83	0.9242	0.02105	0.08047
	Logistic	2.86	0.37	0.9242	0.02105	0.08047
	Normal	2.81	0.51	0.9204	0.04807	0.09484
	Weibull	-5.92	1.92	0.9284	0.01048	0.07734
ANT	Arctangent	1.38	0.74	0.9699	0.04943	0.05037
	Dagum	0.45	3.20	0.9769	0.03771	0.04432
	Error	0.55	0.78	0.9709	0.04047	0.04976
	Gompertz	2.37	0.91	0.9771	0.04046	0.04408
	Gudermannian	1.06	1.49	0.9710	0.04278	0.04963
	Hyperbolic tangent	0.65	0.92	0.9710	0.04176	0.04971

	Logistic	1.41	0.77	0.9710	0.04176	0.04971
	Normal	1.46	1.02	0.9576	0.05999	0.07032
	Weibull	-1.63	0.84	0.9481	0.06346	0.06592
B[a]P	Arctangent	0.98	0.75	0.9126	0.08595	0.08777
	Dagum	0.45	2.45	0.9192	0.07125	0.08753
	Error	0.49	0.57	0.8964	0.08859	0.09894
	Gompertz	1.78	0.89	0.9301	0.06382	0.08029
	Gudermannian	0.98	1.08	0.9038	0.08699	0.09472
	Hyperbolic tangent	0.59	0.67	0.9019	0.08657	0.09625
	Logistic	1.13	0.84	0.9018	0.08669	0.09625
	Normal	1.40	1.36	0.8796	0.07879	0.11685
	Weibull	-1.46	0.99	0.8868	0.08556	0.11456
FLA	Arctangent	2.01	0.59	0.9760	0.03997	0.04460
	Dagum	0.50	7.46	0.9786	0.02885	0.04333
	Error	0.63	1.31	0.9644	0.03745	0.05587
	Gompertz	6.29	1.10	0.9793	0.03037	0.04266
	Gudermannian	1.27	2.60	0.9704	0.03368	0.05093
	Hyperbolic tangent	0.77	1.57	0.9679	0.03479	0.05307
	Logistic	2.05	0.65	0.9679	0.03480	0.05307
	Normal	2.15	1.06	0.9595	0.02965	0.06217
	Weibull	-2.36	0.93	0.9329	0.06307	0.07641

Table S11. (*Continued*).

PAH	SSD model	*a*	*b*	R^2	$RMSE_{50}$	RMSE
FLO	Arctangent	3.20	0.42	0.9811	0.04116	0.03955
	Dagum	0.73	140.77	0.9812	0.02098	0.04143
	Error	1.10	3.51	0.9819	0.01034	0.04156
	Gompertz	138.17	1.67	0.9811	0.02127	0.04149
	Gudermannian	2.04	6.51	0.9830	0.01765	0.03948
	Hyperbolic tangent	1.27	4.05	0.9827	0.01367	0.04013
	Logistic	3.19	0.39	0.9827	0.01367	0.04013
	Normal	3.28	0.74	0.9510	0.03670	0.06584
	Weibull	-6.37	1.86	0.9787	0.03003	0.04498
NAP	Arctangent	3.64	0.33	0.9836	0.04248	0.03627
	Dagum	0.98	2585.31	0.9872	0.04261	0.03432
	Error	1.33	4.85	0.9802	0.04626	0.04285
	Gompertz	2581.68	2.26	0.9872	0.04261	0.03432
	Gudermannian	2.49	9.11	0.9838	0.04279	0.03797
	Hyperbolic tangent	1.54	5.64	0.9825	0.04396	0.03976
	Logistic	3.66	0.32	0.9825	0.04396	0.03976
	Normal	3.68	0.64	0.9669	0.05825	0.05130
	Weibull	-8.03	2.08	0.9687	0.05275	0.05351
PHE	Arctangent	2.56	0.30	0.9779	0.04225	0.04208
	Dagum	0.89	121.25	0.9624	0.05971	0.05684
	Error	1.33	3.37	0.9777	0.04192	0.04413
	Gompertz	119.62	2.06	0.9622	0.06010	0.05702
	Gudermannian	2.58	6.55	0.9804	0.03909	0.04085
	Hyperbolic tangent	1.58	4.00	0.9795	0.04019	0.04201
	Logistic	2.54	0.32	0.9795	0.04019	0.04201
	Normal	2.52	0.53	0.9776	0.04428	0.04449
	Weibull	-6.40	2.36	0.9827	0.03004	0.03953
PYR	Arctangent	1.53	0.46	0.9590	0.07202	0.06231
	Dagum	0.69	8.27	0.9587	0.04602	0.06550
	Error	0.95	1.48	0.9399	0.05866	0.08094
	Gompertz	6.83	1.49	0.9607	0.04482	0.06346
	Gudermannian	1.78	2.76	0.9443	0.06393	0.07658

Hyperbolic tangent	1.10	1.71	0.9425	0.06170	0.07832
Logistic	1.55	0.45	0.9425	0.06171	0.07832
Normal	1.99	1.49	0.7930	0.10242	0.13658
Weibull	-2.85	1.57	0.9188	0.07670	0.09532

Table S12. The fitting parameters (*a* and *b*) and goodness of fit (R^2, RMSE and $RMSE_{50}$) of various SSD models based on the chronic toxicity values (CVs) or ACR-transformed values (CV_{ESS}s) of the four PAHs.

PAH	Function	*a*	*b*	R^2	$RMSE_{50}$	RMSE
B[a]P	Arctangent	0.04	0.65	0.9022	0.10140	0.09201
	Dagum	0.66	1.12	0.9064	0.09497	0.08948
	Error	0.67	0.08	0.9056	0.09577	0.08977
	Gompertz	0.73	1.17	0.9240	0.08142	0.08123
	Gudermannian	1.26	0.12	0.9033	0.09806	0.09081
	Hyperbolic tangent	0.78	0.08	0.9043	0.09700	0.09034
	Logistic	0.11	0.64	0.9043	0.09700	0.09034
	Normal	0.15	0.75	0.8934	0.11813	0.12438
	Weibull	-0.56	1.03	0.8847	0.11149	0.09814
B[a]P*	Arctangent	0.37	0.75	0.9126	0.08593	0.08777
	Dagum	0.47	1.52	0.9112	0.07784	0.09146
	Error	0.49	0.27	0.8964	0.08862	0.09894
	Gompertz	1.04	0.89	0.9300	0.06383	0.08029
	Gudermannian	0.97	0.49	0.9037	0.08709	0.09472
	Hyperbolic tangent	0.59	0.31	0.9018	0.08670	0.09625
	Logistic	0.52	0.84	0.9018	0.08668	0.09625
	Normal	0.80	1.36	0.8796	0.07879	0.11685
	Weibull	-0.86	0.99	0.8867	0.08563	0.11456
FLA	Arctangent	1.89	0.70	0.9298	0.09628	0.07455
	Dagum	0.52	6.50	0.9556	0.07128	0.06060
	Error	0.68	1.29	0.9546	0.08315	0.06132
	Gompertz	5.39	1.12	0.9563	0.06813	0.06015
	Gudermannian	1.26	2.39	0.9484	0.08681	0.06499
	Hyperbolic tangent	0.78	1.49	0.9514	0.08506	0.06324
	Logistic	1.90	0.64	0.9514	0.08506	0.06324
	Normal	1.91	0.77	0.9320	0.11962	0.09467

	Weibull	-2.51	1.09	0.9480	0.09734	0.06555
FLA*	Arctangent	1.36	0.59	0.9760	0.03997	0.04460
	Dagum	0.52	3.95	0.9778	0.02804	0.04420
	Error	0.63	0.89	0.9644	0.03743	0.05587
	Gompertz	3.06	1.10	0.9793	0.03037	0.04266
	Gudermannian	1.27	1.76	0.9704	0.03369	0.05093
	Hyperbolic tangent	0.77	1.07	0.9679	0.03479	0.05307
	Logistic	1.40	0.65	0.9679	0.03479	0.05307
	Normal	1.49	1.06	0.9595	0.02965	0.06217
	Weibull	-1.75	0.93	0.9329	0.06307	0.07641

Table S12. (*Continued*) .

PAH	Function	a	b	R^2	$RMSE_{50}$	RMSE
FLO*	Arctangent	2.62	0.42	0.9811	0.04116	0.03955
	Dagum	0.73	54.66	0.9813	0.02054	0.04134
	Error	1.10	2.87	0.9819	0.01034	0.04156
	Gompertz	52.43	1.67	0.9811	0.02127	0.04149
	Gudermannian	2.04	5.33	0.9830	0.01765	0.03948
	Hyperbolic tangent	1.27	3.32	0.9827	0.01367	0.04013
	Logistic	2.61	0.39	0.9827	0.01367	0.04013
	Normal	2.70	0.74	0.9510	0.03670	0.06584
	Weibull	-5.29	1.86	0.9787	0.03003	0.04498
PHE	Arctangent	2.31	0.65	0.9798	0.05554	0.04086
	Dagum	0.51	10.59	0.9777	0.07147	0.04551
	Error	0.66	1.54	0.9781	0.06198	0.04418
	Gompertz	9.65	1.15	0.9768	0.07444	0.04681
	Gudermannian	1.27	2.96	0.9818	0.05494	0.03995
	Hyperbolic tangent	0.78	1.82	0.9805	0.05739	0.04155
	Logistic	2.33	0.64	0.9805	0.05738	0.04155
	Normal	2.33	0.92	0.9749	0.06383	0.05170
	Weibull	-2.80	1.02	0.9644	0.06866	0.05558
PHE*	Arctangent	1.85	0.30	0.9779	0.04225	0.04208
	Dagum	0.90	29.58	0.9629	0.05872	0.05630
	Error	1.33	2.43	0.9777	0.04192	0.04413

Gompertz	28.06	2.06	0.9622	0.06010	0.05702
Gudermannian	2.58	4.73	0.9804	0.03908	0.04085
Hyperbolic tangent	1.58	2.89	0.9795	0.04019	0.04201
Logistic	1.83	0.32	0.9795	0.04020	0.04201
Normal	1.82	0.53	0.9776	0.04428	0.04449
Weibull	-4.74	2.36	0.9827	0.03004	0.03953

* refer to the models based on the CV_{ESS}.

Table S13. The comparison between the SSD curves based on the acute toxicity value (AV), chronic toxicity value (CV) and ACR-transformed toxicity value (CV_{ES}) from three perspectives.

（a）HC_5

PAHs	AV	CV	CV_{ES}	AV/CV	CV/CV_{ES}	AV/CV_{ES}
BAP	0.26	0.06	0.06	4.24	0.96	4.06
FLA	4.09	3.33	0.78	1.23	4.26	5.22
PHE	28.43	2.14	5.61	13.26	0.38	5.07
FLO	135.79		35.83			3.79

（b）the means of log_{10}-transformed toxicity value.

PAHs	AV	CV	CV_{ES}	AV-CV	$CV-CV_{ES}$	$AV-CV_{ES}$
BAP	1.40	0.15	0.80	1.26	-0.65	0.61
FLA	2.15	2.01	1.49	0.13	0.52	0.66
PHE	2.52	2.33	1.82	0.19	0.51	0.71
FLO	3.28		2.70			0.58

（c）the standard deviations (SDs) of log_{10}-transformed toxicity value

PAHs	AV	CV	CV_{ES}	AV-CV	$CV-CV_{ES}$	$AV- CV_{ES}$
BAP	1.36	0.75	1.36	0.61	-0.61	0.00
FLA	1.06	0.75	1.06	0.31	-0.31	0.00
PHE	0.53	0.92	0.53	-0.40	0.40	0.00
FLO	0.74		0.74			0.00
